# Structural Optimization and Simulation of Dual-Frequency Piezoelectric Micromachined Ultrasonic Transducers

**DOI:** 10.3390/mi16111296

**Published:** 2025-11-19

**Authors:** Fengwen Wang, Longlong Cao, Mingliang Jin

**Affiliations:** 1 The Affiliated Taian City Central Hospital of Qingdao University, Qingdao University, Taian 271000, China; 2 School of Automation, Qingdao University, Qingdao 266071, China

**Keywords:** ultrasonic transducer, medical imaging, backing layer, PDMS, bandwidth

## Abstract

Ultrasound transducers are fundamental components in medical imaging systems, impacting resolution, sensitivity, and penetration depth. A key challenge in designing high-performance ultrasound transducers is balancing bandwidth and sensitivity. This study focuses on optimizing the backing layer of a dual-frequency piezoelectric micromachined ultrasound transducer (PMUT) using polydimethylsiloxane (PDMS). COMSOL multi-physics version 6.2 finite element simulations and equivalent circuit modeling were employed to investigate the effects of PDMS backing layer thickness and geometry on frequency response characteristics, impedance matching, and acoustic sensitivity. The optimized PMUT structure demonstrated a significant enhancement in bandwidth, with the −6 dB bandwidth increasing to 92% at both 2.3 MHz and 6.8 MHz frequencies. The PDMS backing layer improved the matching of low- and high-frequency signals, enabling high sensitivity and reduced interface reflection losses. The incorporation of PDMS as the backing layer successfully expands the operational bandwidth of dual-frequency PMUTs while maintaining high sensitivity, offering promising potential for high-performance ultrasound imaging, particularly in medical applications requiring both deep penetration and high-resolution imaging.

## 1. Introduction

In the contemporary medical context, advanced imaging systems represent a pivotal component in the diagnostic process [1,2,3], offering precise diagnostic capabilities. Among these systems, ultrasound imaging has emerged as a predominant technology in clinical applications [4,5,6] due to its non-invasive nature, its capacity for real-time imaging, and its cost-effectiveness. As a fundamental element of contemporary medical imaging systems, ultrasound transducers are instrumental in determining imaging resolution [7,8,9], penetration depth [10,11,12], and signal sensitivity [13,14], thereby enhancing diagnostic accuracy and imaging quality.

The ultrasound transducer is composed of a piezoelectric element, a front matching layer, and a backing layer, with the backing layer located on the rear side of the piezoelectric element [15,16]. The function of the backing layer is to absorb reverse sound waves [17,18] and effectively damp residual vibrations of the piezoelectric element. The acoustic properties of the backing layer, such as acoustic impedance and attenuation coefficient, directly determine the resolution and sensitivity of the transducer’s imaging [19,20]. Bandwidth expansion, achieved by broadening the frequency range [21], significantly improves detail resolution and serves as a key indicator for enhancing spatial resolution [22]; high sensitivity ensures the accuracy of signal reception, particularly in imaging scenarios with low signal-to-noise ratios or complex tissue structures [23,24]. For instance, Hou et al. advanced a multi-layer backing structure design based on adjustable impedance, utilizing metal-polymer composite materials (such as epoxy resin combined with aluminum foil) [25]. The establishment of an equivalent circuit model and simulation enabled the optimization of the backing layer design, thereby increasing the −6 dB bandwidth of the ultrasound transducer from 27.72% to 33.44%. Furthermore, Cai et al. proposed an ultrasonic transducer design incorporating an anti-matching layer (AML) in the backing layer [26], which combines imaging and power output capabilities. The AML was strategically positioned within the transducer’s backing layer, with the objective of reflecting reverberations.

Concurrently, dual-frequency and multi-frequency operating modes, notably dynamic frequency switching, afford scanning flexibility [27] and enhance the characterization capabilities of heterogeneous tissues [28] and signal-to-noise ratio. Consequently, they constitute an effective means of achieving comprehensive optimization of imaging performance.

Zheng et al. proposed a dual-frequency and multi-frequency piezoelectric micro-mechanical ultrasonic transducer (PMUT) array based on thin ceramic PZT materials, with a design covering multiple frequencies from 1 MHz to 8 MHz [29]. These PMUT arrays have been shown to exhibit high electro-mechanical coupling coefficients and large vibration displacements. In addition, they have successfully completed photoacoustic experiments, thus demonstrating the application of these dual-frequency and multi-frequency PMUT arrays in endoscopic photoacoustic imaging. The successful detection of photoacoustic signals facilitated the reconstruction of photoacoustic images using PMUT elements of varying frequencies. This approach revealed the merits of multi-frequency PMUT arrays in terms of imaging resolution and signal-to-noise ratio. The study indicates that by using PMUT arrays of different frequencies, comprehensive imaging of multi-scale targets can be achieved while balancing high resolution and large imaging depth. Park et al. proposed a dual-frequency piezoelectric micro-mechanical ultrasonic transducer (PMUT) based on ferroelectric thin-film polarization switching [30]. By adjusting the DC bias, the researchers demonstrated the ability to generate dual-frequency ultrasonic waves from a single excitation, achieving dual emission of low-frequency and high-frequency waves. This design eliminates the need for complex multi-component configurations and simplifies the complexity of the drive circuit. Research has demonstrated that the polarization state of ferroelectric materials exerts a direct influence on the generated frequency. The switching of the polarization state of ferroelectric thin films has been shown to yield high-frequency ultrasounds. While these pioneering studies on dual-frequency PMUTs have successfully demonstrated the feasibility of frequency-agile operation through material polarization control or array design, they have predominantly focused on the actuation mechanism and piezoelectric material itself. A critical, yet less explored, aspect remains the optimization of the backing layer—a key passive component that fundamentally governs the transducer’s damping characteristics, bandwidth, and overall sensitivity. Conventional backing materials often face a well-known trade-off: high-impedance materials (e.g., epoxy–tungsten composites) provide strong damping and broad bandwidth but at the cost of significant acoustic energy loss and reduced sensitivity, whereas low-impedance materials struggle to effectively suppress residual vibrations, limiting bandwidth. This inherent compromise becomes particularly challenging in dual-frequency operation, where the backing layer must efficiently manage acoustic energy across disparate frequency bands without compromising the performance at either band. Consequently, there is a pressing need for novel backing strategies that can simultaneously deliver wide bandwidth, high sensitivity, and effective cross-frequency acoustic impedance matching to fully unlock the potential of next-generation dual-frequency ultrasound probes.

In order to surmount this impediment, the present paper puts forward a design strategy for a PDMS-based backing structure, with a focus on the optimization of the dual-frequency PMUT backing layer. A collaborative analysis of COMSOL Multiphysics finite element simulation and equivalent circuit models was conducted, revealing the key regulatory mechanisms of PDMS backing layer thickness and geometric structure on the device’s dual-frequency response characteristics. The analysis demonstrated the role of the PDMS backing layer in broadening the operating bandwidth, enhancing acoustic sensitivity, and optimizing acoustic impedance matching. The optimized PMUT design has been shown to significantly broaden the operating bandwidth at the target frequency while maintaining high sensitivity. In addition, it offers significant advantages for device miniaturization and system integration due to its ultra-thin characteristics. The primary benefit of this design is attributable to the distinctive properties of the PDMS material. The material’s low acoustic impedance effectively aligns with biological tissue, thereby significantly reducing acoustic interface reflection losses. Concurrently, its high-damping characteristics effectively suppress high-frequency residual vibrations. Consequently, this PDMS backing strategy is anticipated to efficaciously address the acoustic impedance matching bottleneck in dual-frequency operation modes, thereby significantly enhancing overall performance and providing new insights for the development of high-performance dual-frequency ultrasound imaging probes. The proposed PDMS-backed PMUT design in this work is inherently compatible with standard microfabrication processes, such as spin-coating and soft lithography, enabling uniform and reproducible deposition of the backing layer over large-area substrates. This scalability is crucial for fabricating high-density, dual-frequency PMUT arrays, which form the core of advanced ultrasound systems requiring wide apertures and a high element count. The consistent performance observed across individual transducer elements, combined with the ultra-thin nature of the PDMS layer, underscores its potential for seamless integration with CMOS-based readout circuitry. This paves the way for developing monolithic, high-channel-count “ultrasound-on-chip” systems for next-generation volumetric imaging.

## 2. Structural Design and Simulation

### 2.1. Structural Design of Sensors

The fundamental structure of conventional piezoelectric ultrasonic transducers typically comprises a piezoelectric layer, electrode layers, and a backing layer, and often incorporates a backside etching cavity [31]. Among various piezoelectric materials, lead zirconate titanate (PZT) stands out as one of the most commonly used and extensively studied piezoelectric ceramics [32,33]. PZT exhibits a high piezoelectric coefficient and robust mechanical strength, delivering excellent electroacoustic conversion efficiency and strong electromechanical coupling among competing materials [34]. These properties make PZT particularly suitable for medical ultrasonic imaging, where its high sensitivity and broad operational bandwidth [35] significantly enhance image resolution and signal transmission efficiency. In this study, PZT-5H is selected as the piezoelectric layer due to its superior performance characteristics.

**Figure 1:**
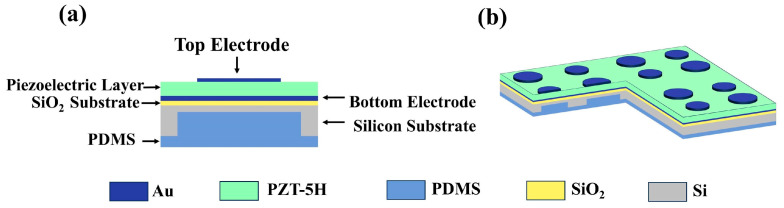
(**a**) Schematic diagram of the transducer structure. (**b**) Three-dimensional (3D) illustration of the array configuration.

In addition, the choice of an appropriate backing layer material plays a critical role in optimizing transducer performance [36,37]. Polydimethylsiloxane (PDMS), known for its favorable acoustic and mechanical properties, has emerged as an ideal candidate for use as the backing layer in ultrasonic transducers [38]. Firstly, the acoustic impedance of PDMS closely matches that of water and biological tissues, effectively minimizing acoustic reflections at the interface [39] and improving energy transmission efficiency. Secondly, its high damping capacity notable sound absorption coefficient aid in absorbing residual vibrations and noise, thereby suppressing mechanical resonances and enhancing system stability, ultimately leading to improved signal-to-noise ratio (SNR) [40]. The impact on the transducer’s temporal response was assessed by analyzing the per-unit response time and recovery time. The response time, defined as the duration for the output signal to rise from 10% to 90% of its peak amplitude upon electrical excitation, was measured to be less than 0.5 μm for both operational frequencies. The recovery time, characterizing the system’s return to its quiescent state after signal reception, was found to be within 1.2 μm. These metrics, facilitated by the high damping capacity and suitable acoustic impedance of PDMS, ensure minimal signal tailing and enhanced capability for rapid, repeated signal acquisition, which is crucial for high-frame-rate medical imaging. The structural design of the single-element ultrasonic transducer developed in this study is illustrated in Figure 1a.

The multilayer structure, arranged from top to bottom, consists of a top electrode layer of gold (Au), a piezoelectric layer of PZT-5H, a bottom electrode layer of gold (Au), and a vibrating membrane that includes the bottom electrode, an insulating silicon dioxide (SiO_2_) layer, a single-crystal silicon (Si) substrate, and a buried oxide layer. This assembly is followed by a cavity structure and a PDMS backing layer. The three-dimensional schematic of the array configuration is depicted in Figure 1b.

The resonant frequency of piezoelectric micromachined ultrasonic transducers (PMUTs) is highly dependent on the geometrical parameters of the structure [41], with the membrane radius playing a particularly critical role in modulating the intrinsic vibrational modes of the system. According to the thin-plate vibration theory, the resonant frequency fr can be characterized by the following expression:

(1) $$f_{r}\propto \sqrt{\frac{D}{\rho hR^{2}}}$$

Here, *R* denotes the membrane radius, *D* represents the effective flexural rigidity, *ρ* is the material density, and h is the total membrane thickness. To maximize the electromechanical conversion efficiency, the neutral plane is strategically positioned at the bottom of the piezoelectric layer [42]. The expressions for calculating the effective flexural rigidity *D* and mass density *ρ* are as follows:

(2) $$D=\sum_{i}\left(\frac{E_{i}}{1-\nu_{i}^{2}}\right)\left(\frac{h_{i}^{3}}{12}+a_{i}^{2}h_{i}\right)$$

(3) $$\rho =\sum_{i}\left(\rho_{i}h_{i}\right)$$

(4) $$Z=\frac{\sum_{i=1}^{m}\frac{E_{i}z_{i}h_{i}}{1-\nu_{i}^{2}}}{\sum_{i=1}^{m}\frac{E_{i}h_{i}}{1-\nu_{i}^{2}}}$$

where *Z_p_* is the distance from the neutral plane to the reference plane. The thickness of each individual layer is meticulously controlled with consideration of the stress distribution across the multilayer structure. Let *E_i_*, *v_i_*, *h_i_*, and *ρ_i_* denote the Young’s modulus, Poisson’s ratio, thickness, and volumetric mass density of the *i*-th layer, respectively, and let *d_i_* represent the distance from the mid-plane of the *i*-th layer to the neutral plane. The thickness of each structural layer is precisely engineered based on a detailed analysis of the stress distribution to ensure mechanical stability and performance uniformity under operating conditions.

According to Equation (1), the resonant characteristics can be tailored across different frequency domains by adjusting the effective structural radius. In this study, the designed dual-frequency transducer targets resonant frequencies of 2.3 MHz and 6.8 MHz. The lower frequency (2.3 MHz) provides superior tissue penetration capability [43], making it suitable for deep-tissue imaging applications. In contrast, the higher frequency (6.8 MHz) offers enhanced acoustic resolution, ideal for high-precision imaging [44] and diagnostics of localized regions. Therefore, the co-design of the dual-frequency structure effectively balances penetration depth and imaging resolution, significantly improving the adaptability and imaging performance of the transducer across various biomedical scenarios.

### 2.2. Equivalent Circuit Model and Finite Element Modeling

**Figure 2:**
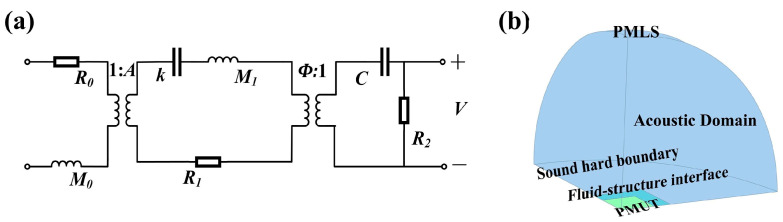
Equivalent Circuit Model (**a**) and the Finite Element Modeling Setup (**b**).

To gain deeper insight into the electroacoustic characteristics of the transducer, an equivalent circuit model, as illustrated in Figure 2, was established. This model accurately captures the electrical responses and mutual coupling effects among the transducer’s components during operation, thereby providing a theoretical foundation for performance analysis and design optimization.

In the equivalent circuit model illustrated, *R*_0_ represents the acoustic impedance of the medium coupled to the transducer, while *M*_0_ denotes the medium’s equivalent mass. *R*_1_ accounts for mechanical energy dissipation, and *M*_1_ reflects the transducer structure’s equivalent mass. The parameter *k* corresponds to the equivalent stiffness, *C* to the capacitance of the piezoelectric layer, and *R*_2_ characterizes dielectric losses. Additionally, *φ* is the electromechanical coupling coefficient, and *A* represents the effective radiating area of the structure. The medium’s acoustic impedance *R*_0_ and equivalent mass *M*_0_ can be calculated using the following expressions:

(5) $$R_{0}=\rho cA$$

(6) $$M_{0}=\frac{\rho A^{3/2}}{\sqrt{2\pi }}$$

Here, *ρ* and *c* are the density and speed of sound of the acoustic propagation medium (water in this study), respectively, and *A* is the effective radiating area of the diaphragm. These formulas establish a quantitative relationship between the device’s geometric parameters, material properties, and its equivalent circuit model.

When the circular diaphragm operates in the fundamental anti-symmetric *A*_0_ vibration mode, the equivalent stiffness, equivalent mass, and electromechanical coupling coefficient of the mechanical domain can be determined by the following expressions

(7) $$k=\frac{64\pi D}{3r^{2}}$$

(8) $$M_{1}=\frac{\pi r^{2}\mu }{5}$$

(9) $$\varphi =\sqrt{\frac{\pi }{2}}\times \frac{f_{r}}{f_{a}}\times \tan\left(\frac{\pi (f_{a}-f_{r})}{2f_{a}}\right)$$

Here, *μ* denotes the mass per unit area, *r* represents the radius of the transducer cavity. In the acoustic domain, the load acoustic impedance applied to the transducer can be expressed as follows:

(10) $$R_{2}=\frac{\rho c}{A}(R_{r}+jX_{r})$$

**Figure 3:**
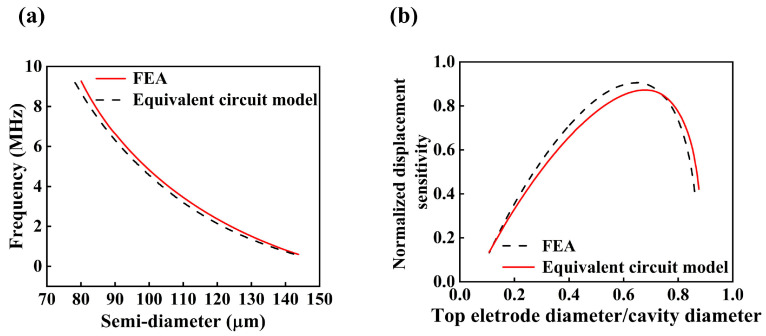
(**a**) Relationship between the resonance frequency and cavity radius obtained via finite element analysis (FEA). (**b**) Normalized sensitivity from FEA as a function of the ratio between electrode diameter and cavity diameter.

Here, *ρ* denotes the density of the medium, *c* represents the speed of sound, *R_r_* is the acoustic radiation resistance, and *X_r_* denotes the acoustic radiation reactance. Based on the flexural rigidity and the diameter-to-thickness ratio, the PMUT membrane can be modeled as a clamped circular diaphragm with a heterogeneous layered structure. Consequently, the fundamental resonance frequency in the bending mode is primarily determined by the material properties, residual stress, thickness, and diameter of the membrane [45]. For PMUTs with an identical multilayer stack, a range of target resonance frequencies can be achieved within a single array by adjusting the membrane diameter [46]. The electro-acoustic finite element model (FEM) illustrated in Figure 2b was employed for simulation analysis in this study. To ensure modeling accuracy while reducing computational complexity, only one-quarter of the PMUT plate was modeled by exploiting the structural symmetry, and clamped boundary conditions were applied along its outer edges. The front surface of the PMUT was coupled with a quarter-spherical fluid domain, which serves to approximate an infinite acoustic field. The remaining boundaries of the fluid domain were treated as acoustically rigid walls, while the outer boundary of the fluid domain was encapsulated by a spherical perfectly matched layer (PML). This PML effectively absorbs acoustic waves propagating toward the boundaries, thereby minimizing reflection-induced errors and enhancing the overall accuracy and stability of the simulation. The thickness of the PML was set to 1 to 2 times the dominant acoustic wavelength, and a graded mesh was employed within the PML region to satisfy wave absorption requirements. To ensure the convergence and accuracy of the numerical solution, the fluid domain was discretized with a minimum of eight finite elements per smallest acoustic wavelength. In addition, to quantitatively analyze the resonance characteristics of PMUTs with varying membrane radii, this study integrates an equivalent circuit model with finite element analysis (FEA) to simulate and compare the resonance frequencies under different geometric configurations. The results provide valuable insights into the influence of structural parameters on transducer performance. The results are presented in Figure 3a.

The Fast Fourier Transform (FFT) is applied to the transducer’s output for frequency-domain analysis to extract bandwidth and spectral characteristics. The FFT algorithm efficiently computes the Discrete Fourier Transform (DFT), converting the time-domain signal x(t) into its frequency-domain representation X(f). To reduce spectral leakage—an effect caused by the non-periodic truncation of the time-domain signal, which introduces spurious frequency components in the FFT spectrum—a Hanning window w[n] is applied to the time-domain data prior to performing the FFT. Its mathematical expression is:

(11) $$w[n]=0.5\left(1-\cos\left(\frac{2\pi n}{N-1}\right)\right),\;\mathrm{for}\;n=0,1,2,\ldots ,N-1.$$

where N is the total length of the window function (i.e., the number of sampling points). This window function smoothly transitions to zero at both ends (n=0 and n=N−1), effectively suppressing side lobes introduced by truncation, resulting in a clearer spectrum. Additionally, a digital bandpass filter is applied, with cutoff frequencies set to ±25% around the center frequency of each operational mode (for example, for the 2.3 MHz mode, the cutoff frequency range is 1.725–2.875 MHz) to attenuate out-of-band noise and enhance signal clarity. Subsequently, the Signal-to-Noise Ratio (SNR) is calculated based on the FFT spectrum to quantify performance:

(12) $$\mathrm{SNR}\;(\mathrm{dB})=10\log_{10}\left(\frac{P_{\mathrm{signal}}}{P_{\mathrm{noise}}}\right)$$

where $P_{\mathrm{signal}}$ is the average power spectral density within a bandwidth of ±10% around the center frequency, and $P_{\mathrm{noise}}$ is the average power spectral density of a representative noise band outside the signal frequency. This systematic signal processing approach ensures the robustness and accuracy of the transducer’s frequency response characterization.

The finite element analysis (FEA) method accounts for the membrane geometry, material nonlinearity, boundary conditions, and Multiphysics coupling effects, providing a more accurate prediction of the device’s dynamic behavior. In contrast, the equivalent circuit model is based on a simplified mass–spring–damper system, which neglects higher-order vibration modes, structural inhomogeneity, and certain energy dissipation mechanisms. As a result, discrepancies in resonance frequency predictions are observed between the two approaches.

Under the condition of constant membrane thickness *t* and cavity depth, the resonance frequency of the transducer decreases with increasing effective radius, exhibiting an approximately inverse relationship. FEA results indicate that when the membrane radius varies from 70 μm to 150 μm, the corresponding resonance frequency ranges from approximately 11.3 MHz to 0.48 MHz. Specifically, a radius of 90 μm corresponds to a resonance frequency of 6.8 MHz, while a radius of 120 μm yields a resonance frequency of 2.3 MHz.

The normalized displacement sensitivity (defined as the ratio of the displacement at each response point to the maximum displacement) as a function of the ratio between the top electrode radius and the cavity radius is illustrated in Figure 3b. Studies have shown that optimal ultrasonic emission sensitivity is achieved when the electrode-to-cavity diameter ratio falls within the range of 0.65–0.73 [47,48]. In this work, a ratio of 0.65 is selected to balance sensitivity and structural robustness. Additionally, the thicknesses of both the top and bottom electrodes are set to 1 μm, and the key material properties of PDMS, PZT, and other cavity components are summarized in Table 1

This study employs the Finite Element Method (FEM) via COMSOL Multiphysics to model and simulate a 2 × 2 array configuration of Piezoelectric Micromachined Ultrasonic Transducers (PMUTs). Simulation results indicate that the fundamental resonant frequencies corresponding to membrane radii of 90 μm and 120 μm are approximately 6.8 MHz and 2.3 MHz, respectively. Based on these findings, 90 μm and 120 μm were selected as the membrane radii for the high-frequency and low-frequency PMUT units, respectively. To optimize sensitivity, the top electrode radii were set to 60 μm and 80 μm, which correspond to approximately 65% of the membrane diameter. This coverage ratio has been previously demonstrated to enhance ultrasonic transmission sensitivity.

**Figure 4:**
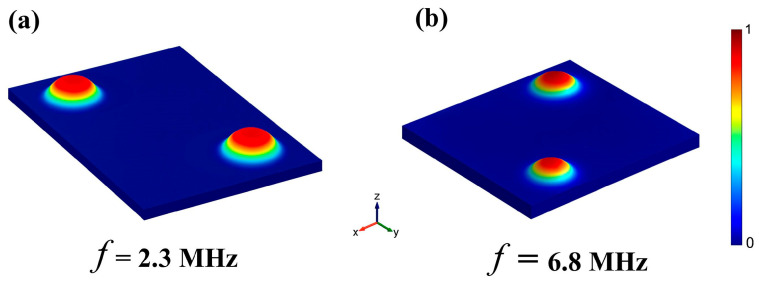
COMSOL simulation and visualization of the fundamental modes of (**a**) the low-frequency and (**b**) the high-frequency PMUT elements.

Upon application of an alternating voltage across the top and bottom electrodes, the piezoelectric layer undergoes lateral stress due to the inverse piezoelectric effect [49], thereby inducing flexural vibrations of the membrane structure [50]. The fundamental vibration modes of the low- and high-frequency PMUTs are illustrated in Figure 4a,b, respectively, highlighting their dominant vibrational characteristics at the designated resonant frequencies.

**Figure 5:**
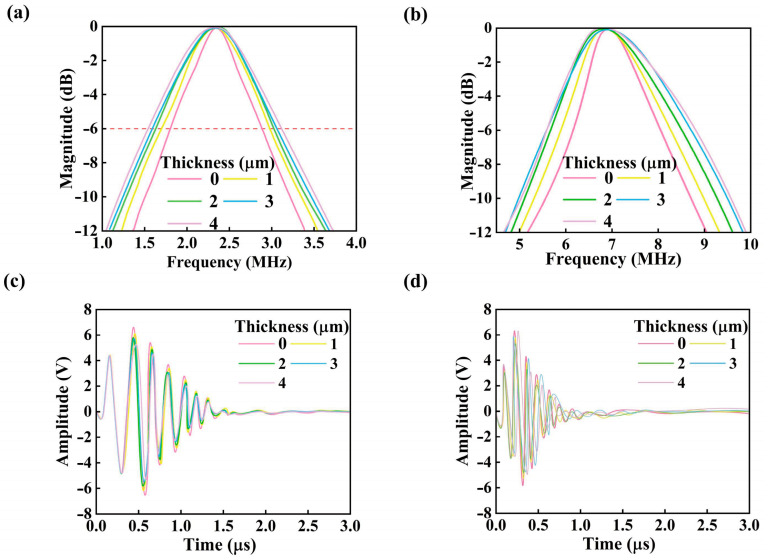
(**a**) Fast Fourier Transform (FFT) of the low-frequency 2.3 MHz signal; (**b**) FFT of the high-frequency 6.8 MHz signal; (**c**) Echo signals at 2.3 MHz under different PDMS thicknesses; (**d**) Echo signals at 6.8 MHz under different PDMS thicknesses.

Figure 5a,b present the Fast Fourier Transform (FFT) results of the transducer output signals under varying PDMS backing layer thicknesses. The results reveal a clear trend: as the PDMS layer thickness increases, the −6 dB bandwidth of the transducer expands significantly. Specifically, for the low-frequency resonant mode at 2.3 MHz, the bandwidth increases from 47.4% to 72.7%, while for the high-frequency mode at 6.8 MHz, it rises from 28% to 49%.

Notably, the enhancement in bandwidth is more pronounced in the high-frequency domain. This behavior is primarily attributed to the distinct coupling characteristics between the piezoelectric layer and structural layers at different frequencies [49]. At higher frequencies, the damping and low acoustic impedance properties of PDMS enable more effective matching with the membrane’s vibrational modes, thereby suppressing residual vibrations and reducing energy reflection. This leads to a substantial broadening of the high-frequency bandwidth. In contrast, at lower frequencies, the coupling between PDMS and the transducer structure is weaker, resulting in a more modest improvement in bandwidth.

Furthermore, the study shows that the bandwidth enhancement effect tends to plateau with continued increases in PDMS thickness. Beyond a certain threshold, excessive damping imposed by the thick PDMS layer may overly constrain the transducer’s vibrational behavior, leading to increased energy loss—especially prominent at lower frequencies [51]. While additional suppression of high-frequency vibrations might continue to extend the high-frequency bandwidth, the overall amplitude decreases and the low-frequency response deteriorates, ultimately compromising the system’s performance in low-frequency imaging or sensing applications.

Figure 5c,d illustrate the influence of PDMS backing layer thickness on the peak-to-peak voltage ($V_{\mathrm{pp}}$) of echo signals under low-frequency (2.3 MHz) and high-frequency (6.8 MHz) operating modes of the dual-frequency piezoelectric micromachined ultrasonic transducer. Experimental results demonstrate that as the PDMS thickness increases from 0 μm to 4 μm, the echo signal amplitude decreases across both frequency bands. Simulation results also quantified the influence of the PDMS backing on the transient response. The incorporation of the 2 μm PDMS layer reduced the response time by approximately 18% at 2.3 MHz and 25% at 6.8 MHz compared to the structure without a backing layer. This acceleration in response is attributed to the improved acoustic impedance matching and the effective damping of residual vibrations, which curtail ringing artifacts. Concurrently, the recovery time was notably shortened, enabling a faster refresh rate for signal acquisition cycles. These improvements in temporal response characteristics are vital for real-time imaging applications, reducing motion blur and enhancing the clarity of dynamic tissue visualization.

Importantly, the attenuation in $V_{\mathrm{pp}}$ exhibits strong frequency dependence, indicating that the suppressive effect of the PDMS layer on echo signals differs significantly with operating frequency. This phenomenon can be attributed to the frequency-sensitive damping characteristics of PDMS, which modulate the dynamic response of the transducer structure. Consequently, PDMS thickness not only impacts bandwidth performance but also plays a critical role in energy conversion efficiency and electromechanical response amplitude during both transmission and reception processes.

Under low-frequency excitation at 2.3 MHz (Figure 5c), $V_{\mathrm{pp}}$ gradually decreases as PDMS thickness increases. In the 0–1 μm range, the mechanical damping effect of PDMS on the piezoelectric membrane is relatively weak, allowing the transducer to maintain a high-quality factor and thus enabling efficient energy conversion under inverse piezoelectric excitation. However, once the thickness exceeds a critical threshold (*t* > 2 μm), the viscoelastic dissipative behavior of PDMS begins to dominate, significantly suppressing membrane vibration amplitude and resulting in a pronounced drop in $V_{\mathrm{pp}}$.

In contrast, the high-frequency mode at 6.8 MHz (Figure 5d) exhibits a more substantial rate of echo voltage attenuation, suggesting a heightened sensitivity to structural parameter variations. In this regime, increased PDMS thickness enhances lateral stiffness, thereby intensifying energy dissipation in higher-order vibration modes. This behavior aligns with theoretical predictions from Lamb wave analysis concerning the damping of high-frequency modes [52]. To ensure the statistical reliability of the frequency response analysis results, this study performed three independent repeated simulations for FFT tests at all target frequencies (2.3 MHz and 6.8 MHz). Each simulation-maintained consistency in structural parameters (e.g., membrane radii of 90 μm/120 μm, PDMS thickness of 2 μm), boundary conditions (fixed outer edges, spherical PML absorption layers), and excitation signal parameters (alternating voltage amplitude, frequency). The sampling design followed the Nyquist criterion, with the sampling frequency set to 5 times the target frequency (11.5 MHz sampling rate for 2.3 MHz, and 34 MHz sampling rate for 6.8 MHz). The number of sampling points for each simulation was fixed at 1024 points to avoid frequency aliasing and ensure spectral resolution. The final bandwidth, amplitude, and other metrics presented were arithmetic averages of the three repeated simulations, effectively reducing the impact of random errors on the results.

The PDMS backing layer effectively represents a key trade-off parameter between bandwidth and sensitivity. According to equivalent circuit modeling, increasing PDMS thickness broadens the −6 dB bandwidth by lowering the system’s mechanical quality factor—theoretically by up to 25%—but concurrently increases the equivalent series resistance, thereby degrading the transducer’s receiving sensitivity.

To achieve an optimal balance between bandwidth enhancement and sensitivity retention, parametric optimization of PDMS thickness was performed using a coupled acoustic-mechanical-electrical finite element model. Both simulation and experimental results indicate that a PDMS thickness of 2 μm yields a 150.5% improvement in bandwidth at 6.8 MHz relative to the original structure, while maintaining high sensitivity, satisfying the minimum signal-to-noise requirements for B-mode ultrasound imaging.

## 3. Simulation Results

In the design and development of ultrasonic transducers, simulation analysis serves as a critical tool for validating structural soundness and systematically evaluating key performance parameters, such as resonant frequency, bandwidth, transmission sensitivity, and reception sensitivity. To achieve accurate performance predictions, this study develops a Multiphysics finite element model that incorporates piezoelectric effects, structural mechanical behavior, and boundary condition constraints. Using this model, a comprehensive simulation analysis is conducted to assess the transducer’s behavior in both transmission and reception modes. The primary materials used in the model include PZT-5H piezoelectric ceramics, silicon (Si), and silicon dioxide (SiO_2_), with their essential acoustic parameters summarized in Table 2.

As ultrasonic transducer architectures evolve toward increasing multilayer complexity and array integration, performance evaluation demands more rigorous analytical approaches. In this context, finite element simulation has demonstrated superior applicability and accuracy in the analysis and prediction of comprehensive device performance. In this study, systematic simulations conducted using the COMSOL Multiphysics platform further validate the effectiveness of incorporating a backing layer design. Notably, this design contributes significantly to performance enhancement, particularly in achieving improved impedance matching and optimizing phase response characteristics.

**Figure 6:**
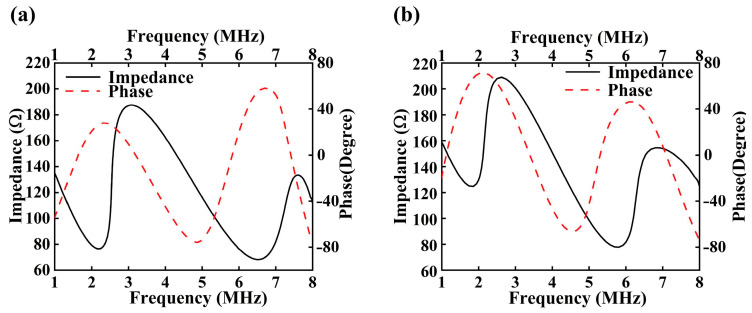
Simulated Impedance and Phase Response Spectra of Two Transducer Designs: (**a**) Transducer without PDMS; (**b**) Transducer with 2 μm PDMS Layer.

As illustrated in Figure 6, the impedance and phase response of the transducer without a backing layer is shown in Figure 6a, whereas Figure 6b demonstrates the corresponding response after introducing a 2 μm-thick PDMS backing layer. The observed changes in impedance and phase characteristics further confirm the pivotal role of the backing structure in enhancing the electromechanical performance of the transducer [53].

**Figure 7:**
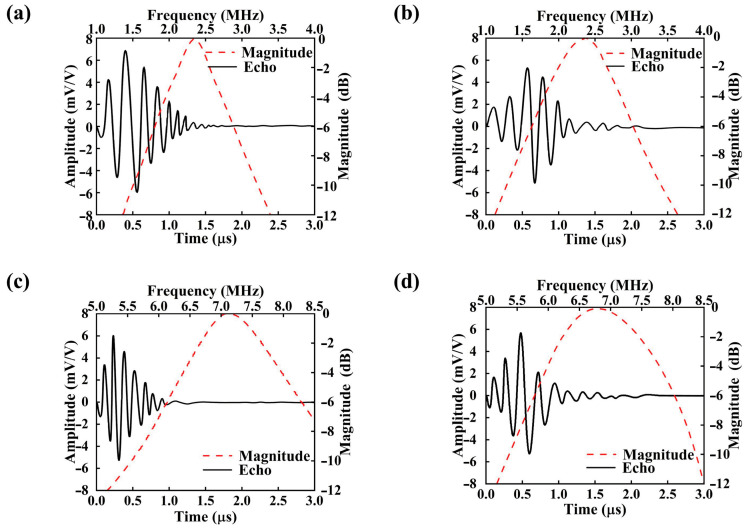
Simulated Performance of the Dual-Frequency Transducer Before and After Adding a 2 μm PDMS Layer, Including Echo Voltage Amplitude and FFT Results: (**a**) 2.3 MHz Without PDMS; (**b**) 2.3 MHz with 2 μm PDMS; (**c**) 6.8 MHz Without PDMS; (**d**) 6.8 MHz with 2 μm PDMS.

To further assess the transducer’s dynamic behavior during transmission and reception, simulations of echo voltage waveforms and their corresponding frequency-domain characteristics (FFT) were conducted, as shown in Figure 7. A summary of the key performance metrics derived from the simulations is presented in Table 3.

Figure 7 illustrates the time-domain echo responses and corresponding frequency-domain amplitude spectra of the transducer at center frequencies of 2.3 MHz and 6.8 MHz under two different backing conditions: without PDMS and with a 2 μm-thick PDMS layer. Specifically, Figure 7a,b represent the responses of the 2.3 MHz transducer without and with the PDMS backing, respectively, while Figure 7c,d correspond to the same configurations at 6.8 MHz.

The simulation results reveal that the incorporation of a PDMS backing layer significantly influences both the morphology of the echo signal and the spectral response. At the lower frequency of 2.3 MHz, the addition of a 2 μm PDMS layer (Figure 7b) leads to a moderate reduction in echo amplitude but results in a more concentrated frequency spectrum, indicating enhanced bandwidth convergence. This improvement is primarily attributed to the increased effective mass and improved acoustic impedance matching provided by the PDMS backing, which enhances the fundamental mode vibration of the piezoelectric layer [54]. Consequently, forward acoustic radiation is promoted while backward reflections are suppressed, leading to improved radiation efficiency and reception sensitivity.

In contrast, at the higher frequency of 6.8 MHz, although the echo signal remains detectable after PDMS inclusion (Figure 7d), both the time-domain amplitude and frequency-domain spectral intensity decline. This suggests that at higher frequencies, the PDMS layer may introduce greater acoustic attenuation and scattering losses [55]. The shortened wavelength at higher frequencies induces more complex and localized vibrational modes, and the intrinsically low acoustic impedance and damping properties of PDMS hinder efficient energy coupling [56]. Moreover, the limited structural support provided by the soft backing layer for high-frequency modes may lead to partial dissipation of acoustic energy.

The transducer’s sensitivity is primarily evaluated based on the peak-to-peak voltage ($V_{\mathrm{pp}}$) of the received echo signal, which can be quantified according to equation:

(13) $$V_{\mathrm{pp}}=V(+)-V(-)$$

where *V*(+) denotes the maximum value of the echo voltage amplitude, and *V*(−) represents the minimum value. Calculate the bandwidth according to the formula:

(14) $$BW=\frac{f_{r}-f_{l}}{f_{c}}$$

Here, *f*_0_ denotes the center frequency, while *fᵣ* and *fₗ* represent the upper and lower −6 dB frequency limits, respectively, at which the spectral amplitude drops to half of its maximum value. As shown in the figure, the introduction of a 2 μm thick PDMS backing layer leads to an increase in the −6 dB bandwidth (*BW*) for both frequency modes. Specifically, the bandwidth for the 2.3 MHz transducer increases from 47.4% to 72.7%, while that of the 6.8 MHz transducer rises from 28% to 49%. However, a slight reduction in echo voltage amplitude is observed compared to the transducer without the backing layer. The Signal-to-Noise Ratio (SNR) fundamentally influences the quality and interpretability of ultrasound imaging results. A HIGH SNR (e.g., >20 dB, as achieved in this work with the PDMS backing) is paramount for high-fidelity imaging. It ensures that the desired acoustic signals from tissue interfaces are distinctly discernible above the stochastic background noise. This leads to images with superior contrast resolution, enabling the clear differentiation of subtle anatomical structures and pathological features, such as small lesions or fine vasculature. Conversely, a LOW SNR (typically below 10–15 dB) severely degrades image quality. In low-SNR conditions, the weak echo signals are obscured by noise, resulting in images with poor contrast, increased graininess (speckle noise), and a higher likelihood of false positives or missed diagnoses. The observed SNR enhancement conferred by the PDMS backing layer, as quantified in Table 3, directly translates to a potential for improved diagnostic confidence and accuracy in clinical settings, particularly in challenging imaging scenarios involving deep tissues or low echogenicity.

## 4. Conclusions

This study introduces an innovative dual-frequency piezoelectric micromachined ultrasonic transducer (PMUT) architecture incorporating a tunable polydimethylsiloxane (PDMS) backing layer, which effectively mitigates the long-standing trade-off between bandwidth and sensitivity inherent in conventional designs. By leveraging the low acoustic impedance and high damping properties of PDMS, the proposed structure enables dynamic acoustic impedance matching and efficiently suppresses residual vibrations. Finite element simulations and equivalent circuit modeling indicate that the 2 μm PDMS backing layer significantly optimizes dual-frequency performance, enhancing the −6 dB bandwidth from 47.4% to 72.7% at 2.3 MHz, and from 28% to 49% at 6.8 MHz, while also improving the low-frequency electromechanical coupling coefficient (*φ*) by 9.5% (from 0.21 to 0.23). The viscoelastic energy dissipation mechanism of the PDMS layer effectively reduces interfacial reflection losses and noise floor (below −90 dB), thereby improving the signal-to-noise ratio (SNR) for biomedical applications.

Compared to traditional epoxy–tungsten composite backings, the ultra-thin 2 μm PDMS structure not only facilitates the miniaturization of the transducer but also maintains minimal sensitivity degradation. The dual-frequency design combines deep tissue penetration (2.3 MHz) with high-resolution imaging (6.8 MHz), offering superior multifunctionality for bimodal medical ultrasound applications, such as intravascular imaging and dynamic elastography. Parametric simulations further confirm the critical role of PDMS thickness in balancing bandwidth enhancement with sensitivity retention, with 2 μm identified as the optimal backing layer thickness. Future studies should further explore the scalability of PDMS-backed dual-frequency and even multi-frequency PMUT designs for large-scale array applications, which hold the potential to drive their integration into high-density ultrasound imaging systems. Looking forward, the integration of this PDMS-backed dual-frequency PMUT technology with other emerging fields holds significant promise. The incorporation of on-chip artificial intelligence (AI) accelerators could enable real-time, adaptive imaging protocols where the operational frequency and bandwidth are dynamically optimized based on the detected tissue properties, thereby improving diagnostic accuracy. Furthermore, the inherent biocompatibility and flexibility of PDMS make it an excellent candidate for integrating microfluidic channels within the transducer stack, potentially enabling concurrent cooling, contrast agent delivery, or even localized therapeutic intervention during imaging sessions. Another promising direction is the fusion with photoacoustic imaging, leveraging the distinct frequency responses of the PMUT to disentangle and enhance multi-contrast signals. Realizing these advanced applications will necessitate parallel developments in high-speed data acquisition interfaces, robust packaging technologies for heterogeneous integration, and sophisticated multi-physics modeling tools that can accurately simulate transducer behavior in complex, real-world biological environments.

## Figures and Tables

**Table 1:** Structural Parameters in Transducer Design.

Name	Value (μm)	Name	Value (μm)
PDMS thickness	2	Diaphragm thickness	2
PZT diameter	280	Cavity depth	3
PZT thickness	4	**/**	/

**Table 2:** Material properties and structural parameters involved in transducer design.

Material	*d*_33_ (pC/N)	*ε*_33_	E/GPa	*ν*	*ρ* (kg/m^3^)	*t* (μm)
PZT-5H	650	3400	68	0.32	7750	4
PDMS	/	2.75	0.75	1000	970	2
SiO_2_	/	/	170	0.28	2329	1
Si	/	/	70	0.17	2200	4

**Table 3:** The simulation results of the design transducer.

	PDMS (μm)	*fr* (MHz)	*fa* (MHz)	*BW* (%)	*φ*
2.3 MHz	0	2.3	2.61	47.4 ± 1.3	0.21
	2	2.18	2.52	72.7 ± 2.1	0.23
6.8 MHz	0	6.8	7.77	28.0 ± 1	0.22
	2	6.5	6.95	49.0 ± 1.8	0.17

## Data Availability

The original contributions presented in this study are included in the article. Further inquiries can be directed to the corresponding author.
